# Social determinants of health and family planning: impact of food and financial insecurity on contraceptive use and pregnancy intention

**DOI:** 10.1017/S1463423625000325

**Published:** 2025-05-16

**Authors:** Breanna Sharp, Covenant Elenwo, Jordan Lowrimore, Caroline Markey, Micah Hartwell

**Affiliations:** 1 Oklahoma State University College of Osteopathic Medicine at Cherokee Nation, Office of Medical Student Research, Tahlequah, Oklahoma; 2 Oklahoma State University Center for Health Sciences, Department of Psychiatry and Behavioral Sciences, Tulsa, Oklahoma; 3 School of Community Medicine, University of Oklahoma - Schusterman Center, Tulsa, Oklahoma; 4 Department of Obstetrics and Gynecology, University of Oklahoma School of Community Medicine, Tulsa, Oklahoma

**Keywords:** contraceptive use, SDOH, family planning, food insecurity, financial instability, pregnancy intention

## Abstract

**Aim:**

In the United States, roughly one million pregnancies occur every year from the misuse and discontinuation of oral contraceptives – which may be affected by an individual’s exposure to social determinants of health (SDOH). For those experiencing poorer SDOH, significant barriers may exist when family planning. Thus, our primary objective is to examine associations between domains of SDOH and contraceptive use as well as pregnancy intention using the Behavior Risk Factor Surveillance System (BRFSS).

**Methods:**

A cross-sectional analysis of 2017 BRFSS was conducted using the SDOH module to examine differences in family planning. We used bivariate and multivariable logistic regression models to measure associations, via odd ratios, between SDOHs and contraceptive use and pregnancy intention controlling for other sociodemographic variables.

**Results:**

We found that individuals experiencing negative SDOH who reported running out of food (AOR: 0.65; CI: 0.50-0.86), were unable to afford balanced meals (AOR: 0.64; CI:0.49-0.84), or had no money left at the end of the month (AOR: 0.45; CI: 0.32-0.64) were less likely to have used contraceptive methods compared to those not experiencing challenges within these SDOH domains. Among women not utilizing contraceptive methods, individuals not intending to get pregnant were more likely to report difficulty affording balanced meals or having financial stability compared to women attempting to become pregnant.

**Conclusions:**

Our study found that the SDOH domains of monthly financial instability and food insecurity are significantly associated with women not using contraceptive measures but not wanting to become pregnant. Addressing barriers to contraceptive access and FP is becoming more important with shifting policies regarding women’s reproductive healthcare. For women seeking contraceptive and FP advice, increased funding may help provide a solution.

## Introduction

Health of an individual is often determined by factors such as genetics, diet, and lifestyle. While these are important, social and economic circumstances further dictate one’s overall health and wellness. The additive effect of all these factors has been termed The Social Determinants of Health (SDOH) – ‘the cause of causes’ (Marmot and Allen, 2014; Marmot and Commission on Social Determinants of Health, 2007). SDOH are the conditions in which people are born, live, and work, that exert substantial influence on the onset and progression of disease (Gurewich et al., 2020). They can be broken up into five pillars: economic stability, education access and quality, health care access and quality, social and community context, and the neighbourhood or built environment in which one lives (SDOH, 2021). These five pillars bring to light the unique role of socioeconomic status (SES) in dictating the quality and quantity of healthcare an individual may receive (Meyer et al., 2014). Those of lower SES often face challenges accessing care as evidenced by the poor outcomes reported when evaluating different measures of health (Smith, 2004). One example is the impact of SES on contraceptive use among women (Iseyemi et al., 2017). Studies show that women of low SES had a higher incidence of contraceptive nonuse or poor contraceptive adherence in comparison to women of higher SES (Iseyemi et al., 2017). Additionally, women with lower SES tend to give birth to more children at a younger age compared to those of higher SES (Larson, 2007; van Roode *et al*., 2017). Given the role of SES on contraceptive use, evaluating the effects of SDOHs on pregnancy and family planning may provide important information for mitigating health disparities among women.

The decision of when to get pregnant is a personal choice that should be protected regardless of an individual’s SES; however, this is not always the case (Parker, 2020). Similar to how SDOH influences an individual’s overall health, socioeconomic conditions have been shown to dictate decisions regarding an individual’s reproductive health and intention on pregnancy. Studies show that women who have had an unintended pregnancy are at an increased risk for maternal depression and delaying prenatal care, while the baby is at increased risk of birth defects and low birth weight. In addition, these babies are reported to experience poor mental and physical health during childhood compared to babies born of intended pregnancies (Family Planning, 2022). Planning when and how to start or expand a family limits the amount of unintended pregnancies and decreases the social and economic ramifications to a mother and her children (Fox and Barfield, 2016). International studies have also examined the relationships of the number of living children in the household and maternal contraception use, with results showing that the number of children in the household often determined the use of some form of contraception (Compton *et al*., 2023; Teshale *et al*., 2023). Therefore, planning to conceive requires an understanding of conception and pregnancy, prenatal care accessibility, social and monetary support, and a safe environment to raise a child.

Unintended and unplanned pregnancies may have devastating effects on any woman who has not had the time, education, or resources to plan for or prevent pregnancy (Woodhams and Gilliam, 2019). Having control over when and whether to use contraception, along with options and education on different contraceptive methods and their proper utilization, serves to mitigate the risk of unintended pregnancies (Forrest, 1994). Facilitating informed decision-making for women requires access to contraceptive education, including education of the different contraceptive methods, their effectiveness, and proper use (Rice et al., 2020). In the United States (U.S.), 65% of women report using some form of contraceptives (Products – Data Briefs – Number 327 - December 2018, 2019; Sawhill and Guyot, 2019). Nevertheless, unintended pregnancies are reported to account for 45% of all pregnancies (Products – Data Briefs – Number 327 - December 2018, 2019; Sawhill and Guyot, 2019). These unintended pregnancies occur primarily among women who practice poor contraceptive compliance such as missing oral contraceptive pills (OCP) or in women who do not use any form of contraception at all (Black et al., 2010). Studies show that approximately 1 million unplanned pregnancies in the US each year result from oral contraception misuse or discontinuation, with the main cause of misuse being missing pills (Black et al., 2010). These women who are most affected by SDOH are the same women who have limited access to safe or affordable abortion options, thus increasing the rates of unsafe abortion practices and stigma surrounding pregnancy termination (Cameron, 2018). Given that individuals negatively affected by SDOH may experience greater barriers in accessing or using contraception methods, and thus face the ramifications of unintended pregnancy, our primary objective was to assess the relationship between SDOH and contraceptive utilization and family planning among women of childbearing age.

## Methods

### Data source

We utilized data from individuals who responded to the 2017 Behavioral Risk Factor Surveillance System (BRFSS), supplied and sponsored by the Center for Disease Control (CDC) and the National Center for Chronic Disease Prevention and Health Promotion. BRFSS collects data in all 50 states, regarding U.S. citizens’ health-related risk behaviours, chronic health conditions, and use of preventative health practices. By collecting information via telephone interview surveys, BRFSS reaches more than 350,000 adults aged 18 and older each year. Being the largest regularly conducted health survey system in the world, data from BRFSS can be used to make decisions concerning health policy, medical intervention and research (About BRFSS, 2019).

### Eligibility and outcome measures

For our outcomes of interest, we used the survey questions, ‘Did you or your partner do anything the last time you had sex to keep you from getting pregnant?’ For the respondents who answered ‘yes’ an additional question asked, ‘What did you or your partner do the last time you had sex to keep you from getting pregnant?’ For the respondents who answered ‘no’ an additional question asked, ‘What was your main reason for not doing anything the last time you had sex to keep you from getting pregnant?’ For each survey question, those who responded ‘don’t know/not sure’ or ‘refused’ were excluded for all outcomes of interest.

### Measures:

#### SDOH

We used the BRFSS SDOH module to assess differences in the utilization of family planning. The SDOH module consists of seven questions that explore the SDOH domains of housing security, neighbourhood safety, food insecurity, financial stability, number of children in the household, and stress. To access housing security, we used the survey question ‘In the last 12 months, how many times have you moved from one home to another?’ To determine neighbourhood safety, we used the survey question ‘How safe from crime do you consider your neighbourhood to be?’ Food insecurity was determined by looking at respondents’ answers to this statement and question: ‘The food that I bought just didn’t last, and I didn’t have money to get more. Was that often, sometimes, or never true for you in the last 12 months?’ For financial stability we assessed the survey questions ‘In general, how do your finances usually work out at the end of the month? Do you find that you usually: end up with some money left over, have enough money to make ends meet, or not have enough money to make ends meet?’ Finally, we assessed respondent stress by examining the question ‘Within the last 30 days, how often have you felt this kind of stress?’ All respondents who answered ‘don’t know/not sure’ or ‘refused’ were excluded from all outcomes of interest.

#### Sociodemographic variables

Sociodemographic variables related to SDOH were extracted from BRFSS to use as controls. These variables included age (18-24, 25-34, 35-44), sex (male or female), race/ethnicity (non-Hispanic White, non-Hispanic Black, Hispanic, or other [including multiple races/ethnicities]), insurance coverage (insured or uninsured), income (less than $10,000; $10,000–$24,999; $25,000–$49,999; or $50,000 or more) and educational attainment (less than high school, high school graduate, some college, or college graduate). Number of children per household was determined from the question ‘How many children less than 18 years of age live in your household?’ For all the sociodemographic variables, respondents who answered ‘don’t know/not sure’ or ‘refused’ were not included in the analysis.

#### Statistical Analysis

We reported sample size (n) and the weighted frequencies (N) and percentages for the demographic characteristics by whether or not the participant was using contraceptive methods. Next, we reported the same statistics (n, N, and %) of individuals having experienced each social determinant of health domain by 1) whether they used contraceptive methods and 2) pregnancy intention (of those who were not using contraceptive methods). We then constructed binary and multivariable logistic regression models to determine the associations, via odds ratios (OR), of SDOH domains and 1) usage of contraceptive methods and 2) pregnancy intention. Statistical analysis was conducted in Stata 16.1 (StataCorp LLC., College Station, TX). Alpha was set at .05 for all analyses. This study was conducted with publicly available data retrieved with no individually identifying information, thus does not meet the requirement of human subject research. The BRFSS data collection procedures were submitted to ethics review and approved through the Centers for Disease Control and Prevention (CDC) (Behavioral Risk Factor Surveillance System, 2021).

## Results

The 2017 BRFSS had an overall response rate of 44.9% per cent. The number of women included in our sample, responding whether or not they used contraceptive measures the last time they had sexual intercourse was 24,454, representing 24,132,367 women in the U.S. Among these respondents, 15081 (N = 15,094,918, 62.55%) reported using contraceptives.

### Sociodemographic results

Results show that usage of contraceptive methods varied by race with women who were American Native/Alaskan Native and Black having the lowest prevalence at 66.1% and 69.7%, respectively, and with women who were White showing highest prevalence at 83.2%. For education, there was a positive correlation between contraceptive use and education level, with 69% of lower-educated women (i.e. did not graduate High School) and ∼84% of women with advanced degrees reporting contraceptive usage. Additionally, we looked at the association with contraception use and the number of children residing in a given household for whom the mother did not intend to become pregnant. Results revealed that of the women who did not have any children living in the household, 19% did not use contraception. For the women who had at least one child living with them, 24% did not use contraception. Among women with two children, 19% did not use contraception, whereas in households with three children, the rate increased to 21% and further to 24% among women who had four or more children living in the household.

### SDOH domain results

Results show that among women who are not using contraceptive methods, food insecurity and financial instability show the greatest statistical significance when compared to women who are using contraceptive methods. Data show that 27.51% of people not using contraception ran out of food while 72.86% of women who use any form of contraception ran out of food. When accessing the women who do use contraceptive methods, 82.49% said they did not run out of food while the remaining 17.51% of women who do not use any form of contraception admitted to running out of food. The ability to afford balanced meals was assessed between women who use contraception and those who do not. Out of the women who reported inability to afford balanced meals, 17.51% of women were not using any form of contraception whereas 82.49% of the women were. Assessment of financial instability was determined by if finances were depleted by the end of the month. Out of those women who answered ‘no’, 18.13% were not using contraceptive methods while 81.87% were using some form of contraception. Comparatively, in women who answered ‘yes’, 36.71% were not using any form of contraceptive methods while 63.29% were. The SDOH domains of neighbourhood safety, stress, housing security, and problems paying the mortgage, rent, or utilities were not significantly different between women who utilize contraceptives and those that do not. Next, we looked at the associations between the SDOH domains and pregnancy intention of individuals not using pregnancy prevention methods. Of the women who reported they ran out of money by the end of the month, we found that 56% had no intention of getting pregnant. In contrast, of the women who reported they did not run out of money by the end of the month, 68% of those women intended on getting pregnant. Of the women who reported they could not afford balanced meals, 52% had no intention of getting pregnant while of the women who could afford balanced meals, 71% planned for a pregnancy. Of the women who reported difficulty paying rent and utility bills in the last 12 months, 54% had no intention for a pregnancy, while of the women who reported no difficulty paying rent and utility bills in the last 12 months, 67% intended for pregnancy. There was a 16% difference in women who planned for a pregnancy in safe (∼66%) and unsafe (50%) neighbourhoods.

### Logistic regression results

Our multivariable logistic regression results showed statistically significant associations between SDOH domains and contraceptive use. Compared to women who reported no to the food insecurity questions, women who reported running out of food (AOR: 0.65; 95% CI: 0.50−0.86) or not being able to afford balanced meals (AOR: 0.64; 95% CI:0.49−0.84) were less likely to have used contraceptive methods. Additionally, compared to those who reported no having their finances depleted by the end of the month, women who reported not having enough money to make ends meet at the end of the month are less likely to report contraceptive use (AOR: 0.45; 95% CI: 0.32−0.64). Domains of neighbourhood safety, stress and housing instability did not show a statistically significant relationship with contraceptive use.

Our multivariable logistic regression results showed statistically significant associations between SDOH domains with no contraceptive use and intention of pregnancy. Compared to women who reported yes to being able to afford balanced meals, those who reported not being able to afford balanced meals were less likely to want a pregnancy (AOR: 0.43; 95% CI: 0.25−0.76). Additionally, compared to women who reported not having their finances depleted by the end of the month, women who reported not having enough money to make ends meet were less likely to want a pregnancy (AOR: 0.44; 95% CI: 0.20−0.97). Finally, compared to women who reported not having problems paying mortgage, rent or utility bills in the last 12 months, women who reported yes to these problems were less likely to want a pregnancy (AOR: 0.45; 95% CI: 0.23−0.89). Domains of neighbourhood safety, running out of food, stress, and frequent moving with no contraception use did not show a statistically significant relationship with pregnancy intention.

## Discussion

The effects of experiencing negative SDOH perpetuate the challenges faced by women of reproductive age and affect decisions regarding contraceptive use and family planning. As shown in Table 1, our study found that the SDOH domain of food insecurity was significantly associated with individuals not utilizing contraceptive measures, and also having no pregnancy intention (among those not using contraception). We also found that as the number of children in the household increased to 4 or more, the percentage of people reporting contraceptive use decreased. Additionally, shown in Table 2, we found individuals who experienced monthly financial instability had a similar association with contraceptive use and pregnancy intention. There were no significant associations observed between contraceptive use and stress, frequent moving, or neighbourhood safety.

**Table 1.** Associations between SDOH domains and contraceptive method use among individuals aged 18 to 40

	Used method to prevent pregnancy	Did not use any contraceptive method	Unadjusted models		Adjusted models	
	n, N, (weighted %)	n, N, (weighted %)	OR (95% CI)	t, P	AOR (95% CI)	t, P
**Neighbourhood safety**						
Safe	1130, 974971 (19.41)	4717, 4048545 (80.59)	1 (Reference)	–	1 (Reference)	–
Unsafe	77, 98562 (29.55)	248, 234999 (70.45)	0.57 (0.36-0.91)	**−2.35, .019**	0.65 (0.41-1.05)	−1.77, .08
**Ran out of food**						
No	887, 695189 (17.51)	3901, 3275311 (82.49)	1 (Reference)	–	1 (Reference)	
Yes	321, 377062 (27.14)	1069, 1012048 (72.86)	0.57 (0.44-0.75)	**−4.1, <.0001**	0.66 (0.50-0.86)	**−3.01, .003**
**Afford balanced meals**						
No	868, 667653 (17.51)	3697, 3145269 (82.49)	1 (Reference)	–	1 (Reference)	–
Yes	341, 410569 (26.41)	1274, 1143846 (73.59)	0.59 (0.46-0.77)	**−3.96, <.0001**	0.65 (0.50-0.85)	**−3.15, .002**
**Ran out of money**						
No	1030, 863771 (18.13)	4524, 3900082 (81.87)	1 (Reference)	–	1 (Reference)	–
Yes	158, 179131 (36.71)	400, 308769 (63.29)	0.38 (0.27-0.54)	**−5.51, <.0001**	0.45 (0.32-0.65)	**−4.32, <.0001**
**Stress**						
No	964, 854259 (19.65)	4072, 3493140 (80.35)	1 (Reference)	–	1 (Reference)	–
Yes	238, 215424 (22.2)	885, 754994 (77.80)	0.86 (0.63-1.16)	−1.00, .32	0.76 (0.57-1.03)	−1.75, .08
**Frequent moving**						
No	1132, 1001097 (19.95)	4652, 4017891 (80.05)	1 (Reference)	–	1 (Reference)	–
Yes	89, 89811 (23.10)	350, 299006 (76.90)	0.83 (0.5-1.37)	−0.73, .46	0.86 (0.51-1.44)	−0.57, .57
**Problems paying bills**						
No	1032, 921859 (19.63)	4386, 3773453 (80.37)	1 (Reference)	–	1 (Reference)	–
Yes	187, 169374 (23.85)	610, 540709 (76.15)	0.78 (0.56-1.08)	−1.49, .14	0.93 (0.67-1.30)	−0.42, .68

n = sample, N = weighted estimate, weighted per cent. Adjusted model controlled for age, race/ethnicity, number of children in the household, and education. Included women were aged 18-40 and answered that they used a contraceptive method (barrier, medical, behavioural, or other) or not, and were not intending to become pregnant or had plausible reason to believe they were not able to get pregnant (infertile, sterilization, hysterectomy, same-sex partner, are currently pregnant or in postpartum period).

**Table 2.** Associations between SDOH domains and pregnancy intention of individuals not using contraceptive methods and between 18 to 40 years of age

	Not intending to get pregnant	Want pregnancy	Unadjusted models		Adjusted models	
	n, N, (weighted %)	n, N, (weighted %)	OR (95% CI)	t, P	AOR (95% CI)	t, P
**Neighbourhood safety**						
Safe	272, 242851 (34.29)	507, 465324 (65.71)	1 (Reference)		1 (Reference)	
Unsafe	18, 35138 (49.56)	36, 35760 (50.44)	.53 (0.20-1.43)	−1.25, .21	0.71 (0.28-1.80)	−0.73, .47
**Ran out of food**						
No	199, 176168 (30.78)	410, 396101 (69.22)	1 (Reference)		1 (Reference)	
Yes	91, 101714 (49.74)	132, 102782 (50.26)	0.45 (0.25-.81)	**−2.69, .007**	0.59 (0.33-1.05)	−1.80, .07
**Afford balanced meals**						
No	193, 155764 (28.64)	395, 388144 (71.36)	1 (Reference)		1 (Reference)	
Yes	98, 123043 (52.14)	147, 112932 (47.86)	0.37 (0.21-0.65)	**−3.45, .001**	0.43 (0.24-0.75)	**−2.95, .003**
**Ran out of money**						
No	245, 215754 (32.25)	493, 453246 (67.75)	1 (Reference)		1 (Reference)	
Yes	41, 58375 (56.13)	48, 45629 (43.87)	0.37 (0.17-0.82)	**−2.46, .014**	0.45 (0.21-0.96)	**−2.06, .04**
**Stress**						
No	231, 221673 (34.72)	451, 416796 (65.28)	1 (Reference)		1 (Reference)	
Yes	59, 56490 (40.82)	90, 81885 (59.18)	0.77 (0.42-1.43)	−.83, .41	0.99 (0.50-1.97)	−0.03, .97
**Frequent moving**						
No	272, 261068 (35.25)	500, 479466 (64.75)	1 (Reference)		1 (Reference)	
Yes	20, 18453 (46.05)	43, 21618 (53.95)	0.64 (0.25-1.60)	−.96, .34	0.68 (0.27-1.70)	−0.83, .41
**Problems paying bills**						
No	239, 226170 (33.11)	484, 456857 (66.89)	1 (Reference)		1 (Reference)	
Yes	52, 51975 (54.12)	58, 44066 (45.88)	0.42 (0.22-0.80)	**−2.65, .008**	0.47 (0.24-0.92)	**−2.19, .03**

n = sample, N = weighted estimate, weighted per cent. Adjusted model controlled for age, race/ethnicity, number of children in the household, and education. Included women were between 18-40 years of age who responded that they did not use contraceptive measures due to wanting to get pregnant (column 1) and the following: didn’t think they were going to have sex/no regular partner, didn’t think about it (using contraception), couldn’t afford birth control or had problem getting it when needed, or had a lapse in the use of a method.

Current literature exploring the impacts of SDOH on other aspects of women’s health show similar findings to our research (Crear-Perry et al., 2021), (Amjad et al., 2019). Women who experience negative SDOH also experience inequalities and limited healthcare access, as summarized in Table 3. This is evidenced by the immense disparity in maternal morbidity and mortality ratios among minority women, especially Black women (Crear-Perry et al., 2021). While different theories have been put forth to explain the observed differences in maternal outcomes between ethno-racial groups, it is important to consider the role of historical and contemporary discrimination from which this disparity likely stems. The deleterious effects of racism have led to generations of educational underachievement, insecure housing, unstable employment, and low SES (Crear-Perry et al., 2021). These cumulative SDOHs have contributed to the perpetual cycle of adverse outcomes among women, especially women of colour. Data showing increases in adverse pregnancy outcomes among teenage mothers can likely be explained by similar burdens of SDOH these young mothers experience (Amjad et al., 2019). Thus, the observed disparities in health care and access to family planning services necessitate efforts to create uniform access to resources across the bounds of class and race. In addition, identifying specific barriers to contraceptive use among groups least likely to use contraceptives can open up avenues for streamlined interventions to meet the needs of different groups of women. In a secondary analysis of the National Survey of Family and Growth, Kavanaugh and Pliskin found that contraceptive use among women who were sexually active and not seeking pregnancy was lowest among 15–24-year-olds (83%) and highest among 25- to 34-year-olds (91%) (Kavanaugh and Pliskin, 2020). As adolescents are often at higher risk of rapid repeat pregnancies because of lack of awareness and misconceptions about return to fertility (Engel *et al*., 2019), improving access to education on proper contraceptive use, and addressing health services provides knowledge gaps and misconceptions about contraceptive service provision might be the first steps in improving contraceptive use among adolescents and possibly, in households with a growing number of children (Chandra-Mouli and Akwara, 2020).

**Table 3.** Demographics of BRFSS respondents between 18 and 40 years of age and use of contraceptive methods

Demographic variable	Not using prevention methods	Using prevention methods	Statistical test, *P*
	n, N, (%)	n, N, (%)	
**Race/Ethnicity**			
White	1892, 1593327 (16.84)	9461, 7867478 (83.16)	X^2^ = 16.21, < .0001
Black	627, 757659 (30.35)	1473, 1738501 (69.65)	
Asian	171, 292112 (24.12)	474, 918726 (75.88)	
American Indian/Alaskan Native	139, 38687 (33.86)	292, 75579 (66.14)	
Hispanic	945, 1146511 (22.65)	2609, 3915507 (77.35)	
Not listed (Non-Hispanic)	172, 105629 (22.51)	569, 363685 (77.49)	
**Education**			
Did not graduate High School	421, 683055 (31.15)	839, 1509397 (68.85)	X^2^ = 21.22, < .0001
Graduated Highschool	1126, 1073058 (22.86)	3114, 3621082 (77.14)	
Attended College/Technical School	1209, 1303290 (20.04)	4678, 5200028 (79.96)	
Graduated from College/Technical School	1179, 852563 (15.84)	6227, 4528349 (84.16)	
**Number of children in the household**			
0	1239, 1282943 (19.17)	5204, 5410488 (80.83)	X^2^ = 3.56, .007
1	947, 989345 (24.07)	2939, 3120428 (75.93)	
2	916, 865192 (19.39)	3702, 3597980 (80.61)	
3	510, 486539 (21.38)	1855, 1789345 (78.62)	
4+	317, 289891 (24.3)	1131, 902921 (75.7)	
**Age**			
M (SD)	29.52 (6.67)	28.74 (6.51)	t = −3.43, .001

n = sample, N = weighted estimate, weighted per cent. Included women either answered that they used a contraceptive method (barrier, medical, behavioural, or other) or not, and were not intending to become pregnant or had plausible reason to believe they were not able to get pregnant (infertile, sterilization, hysterectomy, same-sex partner, are currently pregnant or in postpartum period).

Historically, certain Public Health Organizations were established to help mitigate the obstacles that women of lower SES faced when trying to access family planning. Planned Parenthood – a nonprofit health organization that allows women to make informed decisions about their reproductive health – offers a variety of reproductive and primary health services for women (Our Services, 2023). Planned Parenthood has allowed women who are negatively impacted by SDOH to get the critical reproductive health care they need. However, with the recent *Dobbs* decision overturning *Roe v. Wade*, there is a potential for funding cuts to Planned Parenthood, which would likely negatively impact women and families of lower SES. In Texas, funding cuts to Planned Parenthood led to a 27% increase in births among women who previously had access to injectable contraception, as well as an overall 46% decrease in the number of patients able to access family planning services through Planned Parenthood (Attacks on Access to Care at Planned Parenthood, 2023). Additional publicly supported agencies include safety-net health clinics, federally qualified health centres (FQHC) and health departments which provided critical reproductive health services to 3.5 million low-income women in 2018 alone (Frost et al., 2019). The majority of these women obtained contraceptive care from safety-net clinics and FQHCs, which accounted for 32% and 20% of the population served in 2015 (Frost et al., 2017). In addition, women who obtained contraceptive services from all types of publicly supported providers in 2016 were able to postpone or avoid two million pregnancies that would have been potentially unavoidable without access to publicly supported care (Frost et al., 2019). These unintended pregnancies would have resulted in an estimated one million births and nearly 700,000 abortions (Frost et al., 2019). Thus, expanded funding for nonprofit organizations providing or enhancing access to women’s healthcare, and protecting publically funded reproductive health agencies, could preserve access to necessary reproductive and primary care services for women of low SES.

Federally funded programmes that provide food and other nutritional means for those of lower SES include the Women, Infants and Children (WIC) programme. WIC is a special supplemental nutrition programme that serves pregnant, postpartum, and breastfeeding women, as well as infants and children (up to age five) (Carlson and Neuberger, 2015; WIC Frequently Asked Questions (FAQs), 2023). It provides access to fruits, vegetables, and whole-grain foods while providing infants with formula supplementation (Carlson and Neuberger, 2015; WIC FAQs, 2023). Women who are enrolled in WIC during pregnancy have been shown to give birth to healthier babies compared to women who did not have access to such supplemental services (Carlson and Neuberger, 2015). Given the strict eligibility criteria that guides qualification for WIC, and the limited eligibility window of a child up to five years old, all women affected by multiple domains of SDOH might not be benefiting from these programmes. This could mean more negative outcomes for women of lower SES, including an inability to afford balanced meals, depletion of finances by the end of the month and limited or no options for contraceptive use or family planning. Although services provided by Planned Parenthood and WIC result in positive outcomes for women of low SES, there are still many women who do not have access to proper family planning services. Therefore, it is essential to bridge the gaps created by SDOHs and provide proper access to contraceptives and family planning services for all women of reproductive age.

## Implications/recommendations

Our findings show there is an immediate need to improve contraceptive and family planning access among women negatively affected by SDOHs. The percentage of women who were not engaging in pregnancy prevention, but did not intend to get pregnant, indicates the need for improved contraceptive counselling and sex education among women of reproductive age. Studies using comprehensive risk reduction strategies, including abstinence education, have been shown to be successful. Results included reductions in sexual risk behaviours – such as frequency of sexual activity, number of partners, and frequency of unprotected sexual activity – all while increasing contraceptive use (Chin et al., 2012; Underhill et al., 2008). Therefore, there is a critical need to expand and protect federal funding for public health programmes aimed at addressing reproductive health services. The Title X programme is one that aims to reduce unintended pregnancies and improve contraceptive use and access, especially among low-income and historically vulnerable groups (Institute of Medicine et al., 2009). Women visiting Title X – supported centres may receive critical preventive services, such as pap smear screenings, sexually transmitted disease testing, free contraceptive resources, and family planning counselling (Institute of Medicine (US) Committee on a Comprehensive Review of the HHS Office of Family Planning Title X Program, 2014). Given the recent *Dobbs* decision to overturn *Roe vs. Wade*, and the subsequent reduction in access to legal abortions in the U.S., addressing barriers to family planning and securing federally funded health promotion programmes for reproductive health services is increasingly essential.

Another notable finding associated with contraceptive and family planning nonuse was among the financial insecurity domain of SDOHs, including problems paying rent, mortgage or utilities. Studies show that low-income households are faced with greater energy poverty due to a lack of understanding of energy education and flexible utility billing policies (Hernández and Bird, 2010). It is estimated that low-income households spend 10-20% of their income on energy services compared to high-income households at only 5% (Wong-Parodi et al., 2013). Expanded funding and coordinated regional approaches for optimizing weatherization services and financial assistance under the Low Income Home Energy Assistance Program (LIHEAP) might provide a solution. LIHEAP provides eligible households with funds to pay their utility bills during the coldest and hottest months of the year. Additional funds are available to return service to eligible households at risk of losing their utilities (LIHEAP, 2023). Securing access and funding for LIHEAP and similar utility assistance programmes may alleviate the influence of utility insecurity on family planning and contraceptive use decisions. In addition, rental assistance services provided under the U.S. Housing and Urban Development (HUD) must be increasingly funded, alongside efforts to mitigate supply constraints for subsidized rental services (Fischer and Sard, 2013; Keene et al., 2020). As HUD is a primary source of affordable housing for low-income families, reassessing the poverty guidelines that dictate eligibility for rental assistance is crucial (Keene et al., 2020). Further efforts to expand eligibility for nutrition and energy assistance services might alleviate the impacts of food and financial insecurity on contraceptive use and family planning decisions among women of reproductive age.

## Limitations

Limitations to this study included that the BRFSS survey poses a limitation in that it is self-reported data, which may include implicit bias. The BRFSS dataset did not include a survey question asking when the last time the reporting individual had sexual intercourse nor specify whether or not the individual had sexual intercourse in the last 12 months. In consideration of these limitations, the advantage of using BRFSS is that it is a large nationally representative dataset with more than 350,000 adults being surveyed annually, which would likely minimize self-report bias. Given that our study was cross-sectional in nature, the results are correlational rather than causal and should be interpreted as such. Future researchers may consider more advanced methods to determine causal pathways such as a longitudinal cohort study to assess the interplay of SDOH and FP and how they evolve with time.

## Conclusions

Our study found that the food insecurity and monthly financial instability domains of SDOH were significantly associated with lack of contraceptive use and having no intention of pregnancy (among those not using contraceptive measures). With changing policies around women’s reproductive healthcare, addressing barriers to family planning and contraceptive access is increasingly critical, especially as the number of children in the household increases. Expanded funding for public health programmes, revised eligibility guidelines for nutrition assistance programmes, and streamlined interventions for specific groups of women may provide solutions for women seeking contraceptive and family planning services.
